# Transcriptome profiling analysis reveals metabolic changes across various growth phases in *Bacillus pumilus* BA06

**DOI:** 10.1186/s12866-017-1066-7

**Published:** 2017-07-11

**Authors:** Lin-Li Han, Huan-Huan Shao, Yong-Cheng Liu, Gang Liu, Chao-Ying Xie, Xiao-Jie Cheng, Hai-Yan Wang, Xue-Mei Tan, Hong Feng

**Affiliations:** 1 Key Laboratory of Bio-resources and Eco-environment, Ministry of Education, Sichuan Key Laboratory of Molecular Biology and Biotechnology, Sichuan University, Chengdu, 610064 Sichuan People’s Republic of China; 2 College of Life Sciences, Sichuan University, Chengdu, 610064 Sichuan People’s Republic of China

**Keywords:** *Bacillus pumilus*, Bacterial flagella, Chemotaxis, Gene expression, Gene regulation, Protease, RNA-seq, Sporulation, Stress response, Transcriptome, Tricarboxylic acid cycle

## Abstract

**Background:**

*Bacillus pumilus* can secret abundant extracellular enzymes, and may be used as a potential host for the industrial production of enzymes. It is necessary to understand the metabolic processes during cellular growth. Here, an RNA-seq based transcriptome analysis was applied to examine *B. pumilus* BA06 across various growth stages to reveal metabolic changes under two conditions.

**Results:**

Based on the gene expression levels, changes to metabolism pathways that were specific to various growth phases were enriched by KEGG analysis. Upon entry into the transition from the exponential growth phase, striking changes were revealed that included down-regulation of the tricarboxylic acid cycle, oxidative phosphorylation, flagellar assembly, and chemotaxis signaling. In contrast, the expression of stress-responding genes was induced when entering the transition phase, suggesting that the cell may suffer from stress during this growth stage. As expected, up-regulation of sporulation-related genes was continuous during the stationary growth phase, which was consistent with the observed sporulation. However, the expression pattern of the various extracellular proteases was different, suggesting that the regulatory mechanism may be distinct for various proteases. In addition, two protein secretion pathways were enriched with genes responsive to the observed protein secretion in *B. pumilus*. However, the expression of some genes that encode sporulation-related proteins and extracellular proteases was delayed by the addition of gelatin to the minimal medium.

**Conclusions:**

The transcriptome data depict global alterations in the genome-wide transcriptome across the various growth phases, which will enable an understanding of the physiology and phenotype of *B. pumilus* through gene expression.

**Electronic supplementary material:**

The online version of this article (doi:10.1186/s12866-017-1066-7) contains supplementary material, which is available to authorized users.

## Background

*Bacillus pumilus* is an endospore-forming, gram-positive, rod-shaped bacterium. Due to its metabolic diversity and spore dispersal, *B. pumilus* is ubiquitous in various environments and commonly resistant to extreme environmental conditions [1, 2]. Similar to other *Bacillus* species, *B. pumilus* is able to secrete a large number of industrial enzymes, such as lipases [3], xylanases [4], and proteases [5–7]. Therefore, *B. pumilus* has attracted attention in biotechnology and was selected to engineer novel industrial production strain [8, 9]. In addition, some strains of *B. pumilus* were used to produce valuable small molecules [10, 11] and have served as biocontrol agents to manage plant diseases [12]. However, studies pertaining to the physiological and metabolic processes of *B. pumilus* are extremely limited in comparison with the other *Bacillus* species.

Along with the great advances in genomics, many strains of *B. pumilus* have been selected for genome sequencing. To date, more than 30 genomes of *B. pumilus* are available at NCBI. Based on genomic alignments, *B. pumilus* is closer to *B. subtilis*, *B. licheniformis*, and *B. amyloliquefaciens* [13], suggesting that similar metabolic or physiological processes may exist between *B. pumilus* and the model organism *B. subtilis*. Although the genome is considered the blueprint of life, much information regarding the physiological or metabolic processes is not directly accessible from the genome [14]. Therefore, various omics technologies such as transcriptomics, proteomics and metabolomics, have been employed as essential steps toward gaining insights into cell physiology from the genome. For instance, a combined omics-based approach has been applied to *B. pumilus* to try to understand cell physiology in response to oxidative stress and protein secretion [15, 16]. For this purpose, RNA-seq -based transcriptomics analysis is one of the most powerful tools that not only provides important insights into the functional elements of the genome, gene expression patterns and regulation [17], but also offers a simpler and more cost-effective approach [18]. Therefore, the RNA-seq method has been widely applied to many *Bacillus* species. For example, *B. subtilis* [19, 20], *B. licheniformis* [21], and *B. thuringiensis* [22] were examined by RNA-seq analysis.

*B. pumilus* BA06 has been isolated from proteinaceous soil and is demonstrated to be able to secrete extracellular proteases that exhibited great potential in leather processing [5, 23, 24]. However, the production of extracellular proteases is considered to occur at the stationary growth phase and is extensively regulated in many species of *Bacillus* [25, 26]. For example, nitrogen and carbon sources have great impacts on the production of extracellular proteases [27]. Thus, medium components have usually been optimized for fermentation of proteases [28]. In addition, the other physiological processes of secondary metabolite synthesis, protein secretion, and sporulation occur during the stationary growth phase [29–31]. Based on studies in *B. subtilis*, a transition point occurs between the exponential growth and stationary growth phases [32]. Over the various growth phases, the expression of many genes may be turned off and another set of genes may be turned on. For example, more than 100 genes, whose expression was induced at the onset of the stationary growth phase, have been assigned to the SigB regulon [33, 34].

By taking advantage of the RNA-seq technology, a time-resolved transcriptomic analysis to cover the various growth stages of *B. pumilus* BA06 was performed. The results clearly indicated that changes in gene expression or metabolic pathways were occurring during the various growth phases; this observation will be helpful for us toward understanding the physiology and phenotype of *B. pumilus*.

## Results

### Cell growth, sporulation and extracellular protease activity

**Fig. 1:**
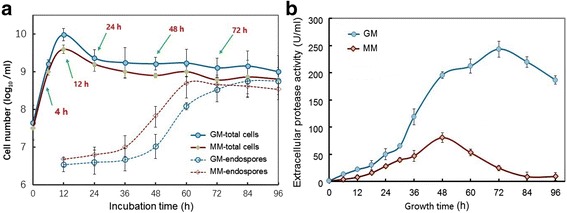
Growth curve (**a**) and extracellular proteolytic activity (**b**) of *B. pumilus* BA06 in the minimal medium (MM) and MM plus gelatin (GM)

To gain insights into the temporal transcriptome changes of *Bacillus pumilus* BA06, two sets of cultures were established in 50 -ml of MM (minimal medium) and GM (MM plus 2% gelatin) in 250 ml flasks. Initially, the growth curve was monitored by measurement of OD_600_ at 6-h intervals (data not shown). Unexpectedly, the cell density suddenly declined after 12 h. To reflect the intrinsic cell growth, the numbers of vegetative cells and endospores in the same cultures were further calculated by plate -counting. Fig. 1a shows the growth curves of the total cell and endospore counts at various time points; it is evident that the total cell number arrived at its peak at 12 h and then declined at 24 h. The addition of gelatin to the medium did not change the pattern of cell growth except that the cell number was slightly higher. However, endospore formation started at approximately 24 h, and then increased greatly up to a peak at 60 h and 72 h in MM and GM, respectively. Therefore, the cell growth of *B. pumilus* BA06 could be obviously divided into two phases: the exponential growth and stationary phases with a transition point at 12 h under this condition [32].

Meanwhile, the extracellular protease activity was also monitored during the growing period. Fig. 1b shows that the extracellular protease activity mainly occurred at the onset of the stationary phase with a peak at 48 h in MM, which was consistent with previous results [25]. Moreover, the addition of gelatin led to a higher peak at the delayed time (72 h) in extracellular protease activity (Fig. 1b), indicating that the nitrogen source (gelatin) affected the production of extracellular proteases.

### RNA sequencing and identification of differentially expressed genes (DEGs)

Based on the growth curve, cell samples were collected at 5 time points (4 h, 12 h, 24 h, 48 h, and 72 h) across the exponential growth phase, transition point, and early and late stationary growth phases from three independent cultures of MM and GM; and total RNAs were subsequently isolated (Additional file 1). Three RNA samples for each time point were mixed equally, and used for Illumina sequencing. Finally, approximately 1.5 G clean data were generated for each time point. The summary of the sequence data is presented in Table 1. It was found that more than 98% of the clean reads from each sample could be mapped to the reference genome, indicating that the transcriptome data were sufficient for further analysis.

**Table 1:** Summary of RNA-seq and the reads mapped to the genome of *B. pumilus* BA06

Sample name	Left size (M)	Right size (M)	Number of reads	Mapped reads (%)	Paired reads (%)	GC (%)
MM-04	866.19	875.33	23,415,852	23,010,464 (98.27)	23,005,388 (98.27)	42.37
MM-12	847.89	854.26	24,131,710	24,009,599 (99.49)	24,009,599 (99.49)	42.49
MM-24	729.02	738.72	20,629,536	20,492,441 (99.34)	20,492,441 (99.34)	42.77
MM-48	755.99	761.63	20,882,864	20,732,600 (99.28)	20,732,600 (99.28)	42.27
MM-72	829.13	834.27	23,020,742	22,782,607 (98.97)	22,782,607 (98.97)	42.40
GM-04	770.57	777.42	21,326,144	21,223,188 (99.52)	21,223,188 (99.52)	42.33
GM-12	888.51	898.43	25,386,720	25,258,250 (99.49)	25,258,250 (99.49)	42.67
GM-24	792.57	799.42	9,023,902	8,970,697 (99.41)	8,970,697 (99.41)	43.21
GM-48	796.46	803.54	17,332,139	17,212,840 (99.31)	17,212,840 (99.31)	42.61
GM-72	749.20	753.69	20,854,598	20,625,390 (98.9)	20,625,390 (98.9)	42.22

**Fig. 2:**
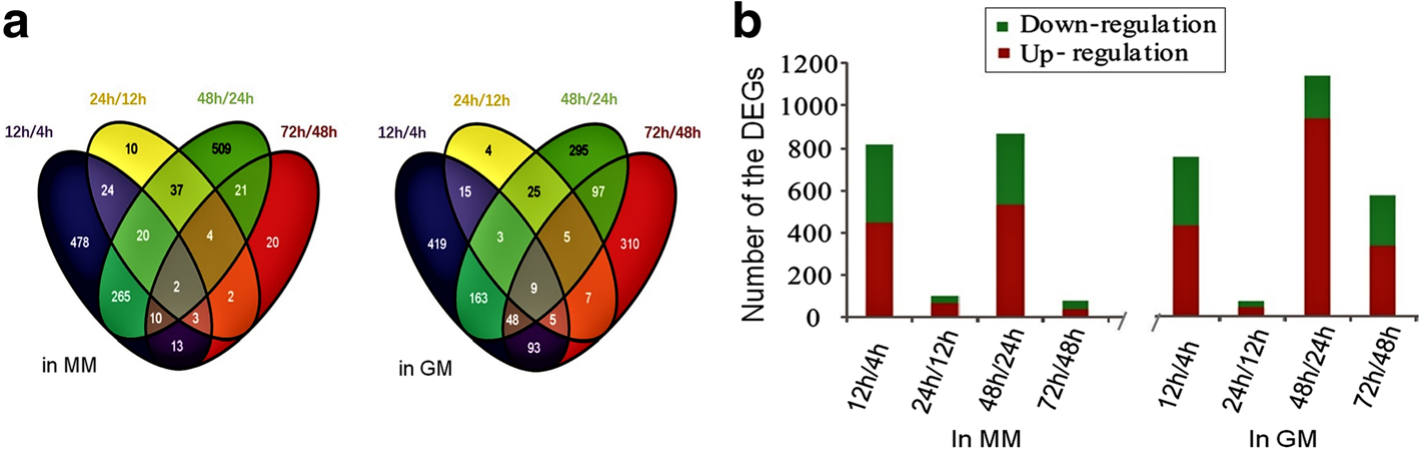
Analysis of the differentially expressed genes of *B. pumilus* BA06 grown in MM and GM over the entire growth curve. **a** Venn diagram analysis and (**b**) numbers of DEGs between toe time points

Based on the number of mapped reads against the genome with the Bowtie software, the expression level of each gene was calculated in terms of the FPKM value (A complete list of annotated genes with the FPKM value is presented in Additional file 2.). The DEGs were extracted using the edgeR software with *p* value <0.05 and log_2_(fold-change) > 1. Consequently, 1418 and 1499 DEGs were identified between the two different time points in MM and GM, respectively. The numbers of DEGs between the various time points during the growth course of *B. pumilus* BA06 were analyzed using a Venn diagram (Fig. 2a). Globally, two striking changes of the DEG numbers between the two time points could be observed in the MM cultures, one for the transition point (12 h) and one for the stationary growth phase (48 h). There were 815 and 868 DEGs identified between 12 h/4 h and 48 h/ 24 h (Fig. 2a), respectively, indicating that the metabolic transition and endospore development were an intrinsic consequence of the gene expression change. However, the addition of gelatin to the MM did not change the gene expression pattern of *B. pumilus* BA06 significantly, except that more DEGs were also found between 72 h/48 h (Fig. 2b).

All the DEGs were categorized by the RAST system [35]. Table 2 shows the top 12 subsystems with the numbers of the involved DEGs. The most abundant subsystems of DEGs were related to metabolism of “Carbohydrates”, followed by “Amino Acids and Derivatives”, which suggested that the changes in primary metabolism were great between the various growth phases. In addition, the DEGs involved in “Mobility and Chemotaxis” and “Dormancy and Sporulation” were also great in number (see details in the following sections), indicating that the physiology or phenotype would be changed greatly. It was noticed that the change in “Iron Acquisition and Metabolism” would be great for this bacterium since the expression levels of many genes included in iron metabolism were altered between the different growth phases. For example, several operons responsive to iron compound uptake (peg.2296–2299) and siderophore biosynthesis (peg.34–39) were up-regulated upon entry into the transition point (12 h) (Additional file 2). In fact, the operon encoding siderophore biosynthesis is missing in model organism *B. subtilis* 168, suggesting that something in the physiological or metabolic processes is different among the various *Bacillus species.*

**Table 2:** Top 12 functional classification of the DEGs of *B. pumilus* BA06 during the entire growth curve by RAST analysis

Subsystems	in MM				in GM			
	12/04	24/12	48/24	72/48	12/04	24/12	48/24	72/48
Carbohydrates	98	2	45	5	79	1	37	22
Amino Acids and Derivatives	42	2	44	1	45	8	29	12
Motility and Chemotaxis	55	7	55	5	55	10	34	33
Cofactor/Vitamin/Prosthetic Group	28	4	12	0	28	1	15	23
Clustering-based Subsystems	25	0	28	0	27	1	42	17
Dormancy and Sporulation	20	0	59	0	16	0	34	31
Cell Wall and Capsule	16	1	17	2	14	0	10	16
Iron Acquisition and Metabolism	18	2	9	0	22	0	14	14
Stress Response	16	0	13	2	13	0	15	13
Nucleosides and Nucleotides	16	1	4	1	16	0	5	7
Protein Metabolism	11	1	14	1	10	0	17	8
Respiration	12	0	6	0	12	0	4	2

### Changes in the metabolic pathways at the transition point of growth

**Fig. 3:**
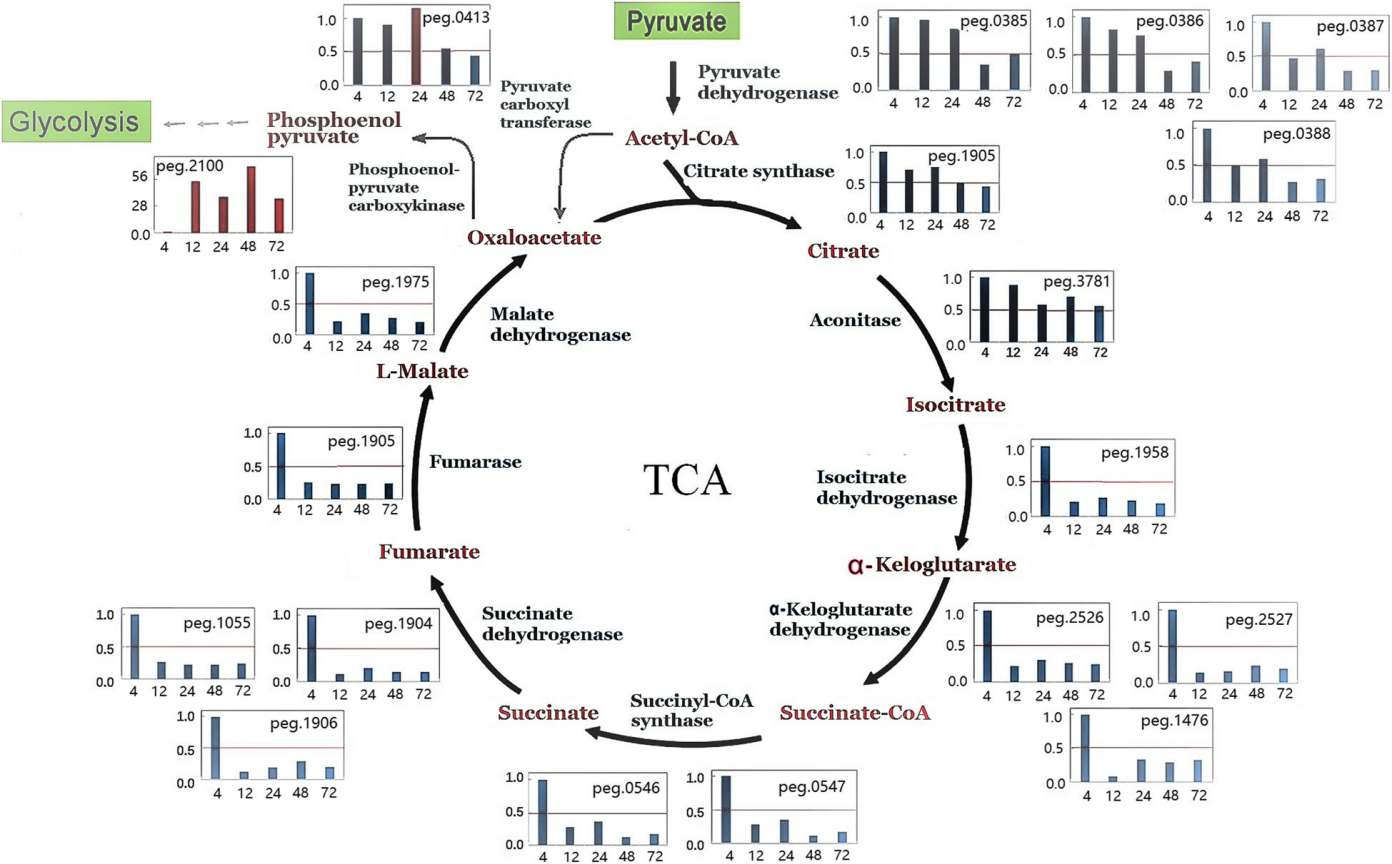
Down-regulation of the tricarboxylic acid cycle of *B. pumilus* BA06 grown in MM. The gene expression level of each gene was normalized to 1.00 for the first -time point (4 h)

A transition point is recognized as a growth phase where the cells cease exponential growth and enter the stationary growth phase in *Bacillus* [32]. Previous studies have revealed that the expression levels of many genes involved in various metabolic pathways could change between the various growth phases in *B. subtilis* [36, 37]. Similarly, a large number DEGs in *B. pumilus* BA06 were identified at the transition point (12 h) from exponential growth in both cultures of MM and GM (Fig. 2). By KEGG analysis, several metabolic pathways were significantly enriched. First, the tricarboxylic acid cycle (TCA) pathway was down-regulated, since the expression levels of almost all the genes involved in the TCA cycle were decreased significantly (Fig. 3). There was no large difference for the cells cultivated in MM and GM (Additional file 3). However, the metabolic pathway from acetyl-CoA to phosphoenol-pyruvate via oxaloacetate was up-regulated [38], which may provide sufficient substrate for the downstream pathway of glycolysis. It was noticed that the accompanying respiration metabolism was also down-regulated (Additional file 3).

**Fig. 4:**
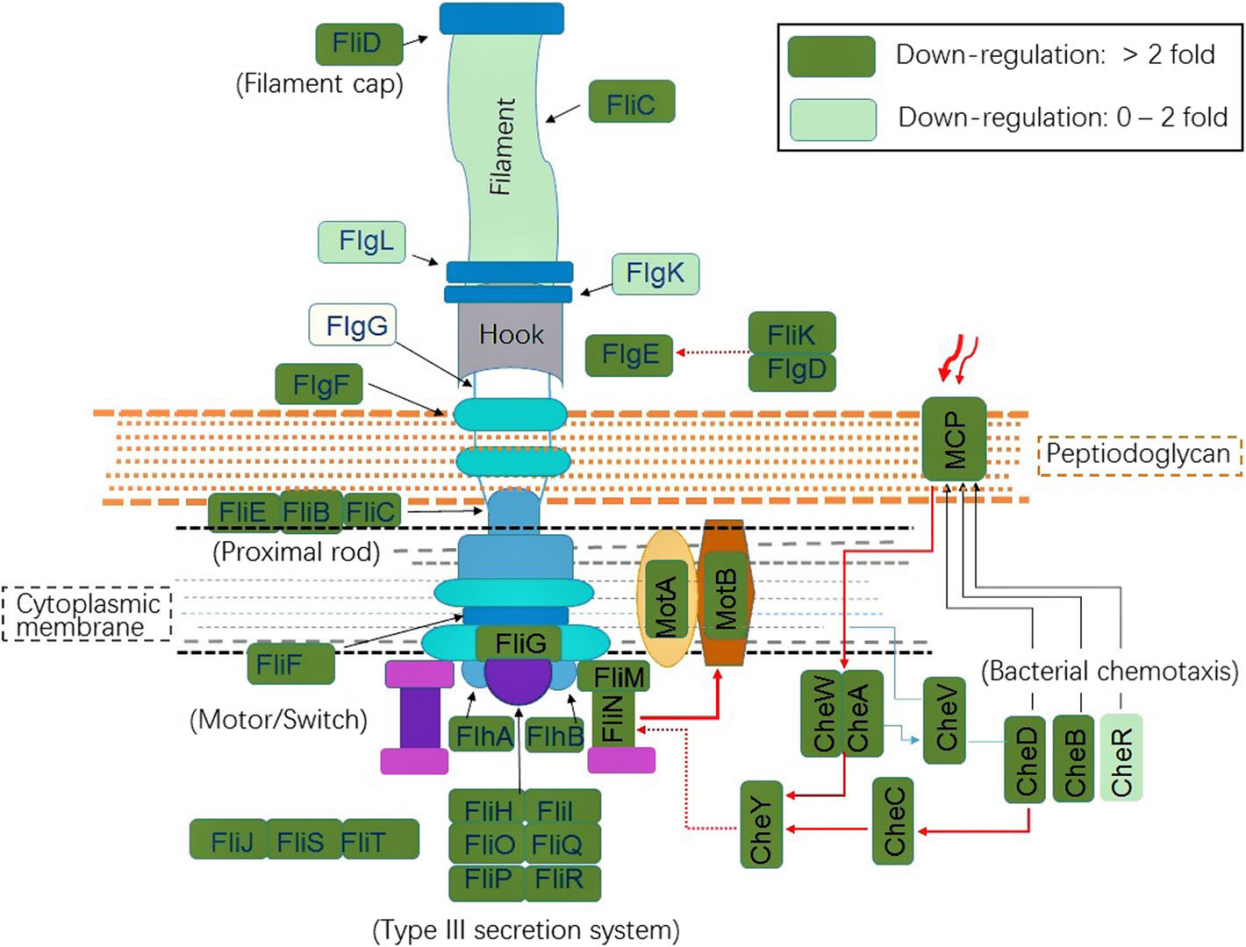
Down-regulation of genes involved in flagellar assembly and the chemotaxis signaling pathway of *B. pumilus* BA06 grown in MM medium at the transition from the exponential growth phase

Second, down-regulation of genes encoding flagellar structural proteins and genes involved in chemotaxis signaling was also observed (Fig. 4), indicating that the swarming mobility of *B. pumilus* BA06 may decrease when entering the transition point. In *B. subtilis*, the sigma factor (*sigD*) and the *swrA* gene were responsive toward activating the expression of the flagellar operon [39]. Similarly, the homologs encoding sigD (peg.585) and swrC (peg.2650) were identified in *B. pumilus* BA06, which were demonstrated to be down-regulated at the transition point (Additional file 4). For swarming mobility, biosynthesis of biosurfactin is also necessary [40]. The expression of the *srf* operon, which encodes genes (peg.2905–2910) for the biosynthesis of biosurfactin in *B. pumilus*, was also down-regulated (Additional file 4). Altogether, the swarming mobility of BA06 may be seriously attenuated when the cells enter the transition point and thereafter.

### Continuous up-regulation of sporulation-related genes

**Fig. 5:**
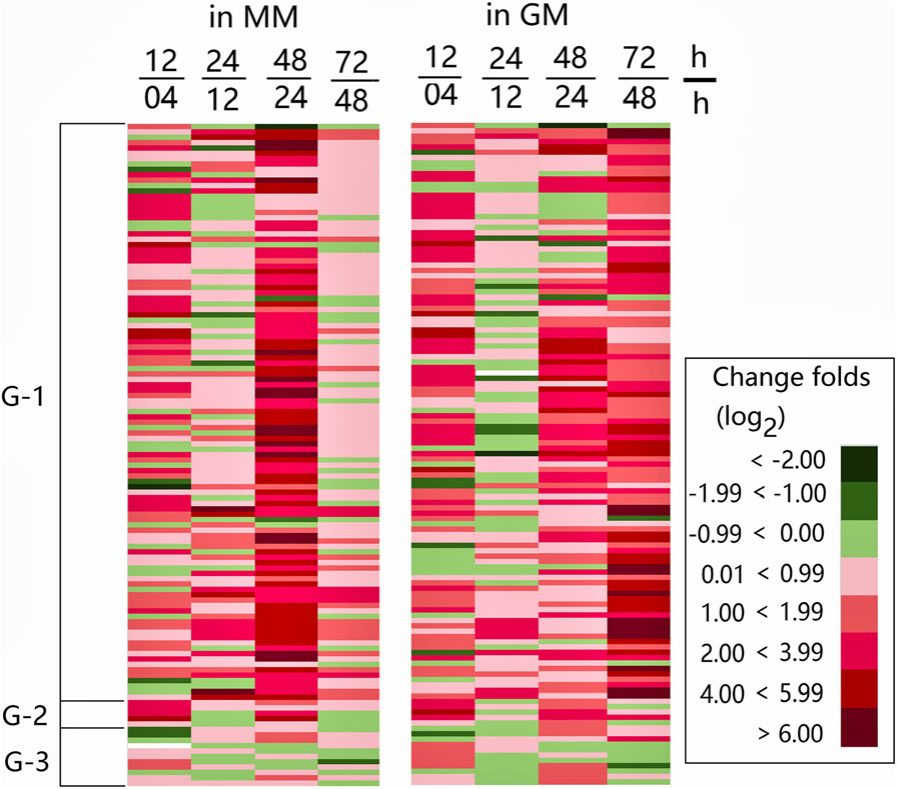
Clustering analysis of the expression changes to genes involved in sporulation of *B. pumilus* BA06. G-1 represents the sporulation-related genes; G-2 displays the genes encoding the sporulation kinases and sporulation initiation phosphotransferases that are involved in sporulation initiation; G-3 shows five regulatory genes encoding sporulation sigma factors SigE, SigF, SigG, SigK, and SigH

For *Bacillus* species, a critical characteristic is the formation of the endospore, a kind of dormant cells with high resistance to environmental stress. Endospore development starts at the onset of the stationary growth phase, which is subject to highly hierarchical regulation [41]. By means of RAST annotation and manually searching, 123 genes were categorized into “Dormancy and Sporulation” (Additional file 5). A cluster analysis of all the sporulation-related genes is shown in Fig. 5, which was divided into three groups (G-1, G-2 and G-3). A great number of the genes in group G-1 displayed a sharp surge in expression at 12 h and then at 48 h in MM. However, the addition of gelatin to the MM led to an extra surge in expression at 72 h, which was consistent with the observation that endospore formation was delayed in GM in comparison with MM (Fig. 1b).

A master regulator protein, Spo0A, governs sporulation initiation through phosphorylation by a series of kinases and phosphotransferase [39]. All of these genes were identified in *B. pumilus* BA06 (Additional file 5) and were clustered into group G-2 (Fig. 5). The expression profile of these genes was generally different from the G-1 group. Many of these genes (G-2) were repressed at the later growth phases, perhaps indicating their leading role only in sporulation initiation.

During the procedure of endospore formation, four sigma factors (SigE, SigF, SigG, and SigK) play important roles in *B. subtilis* [41]. Their counterparts in *B. pumilus* BA06 were identified (group G-3), and all of their expression levels were continuously up-regulated across all growth phases (Fig. 5 and Additional file 5). In contrast, expression of *sigH* (peg.2382) fluctuated over the growth course.

### Extracellular proteases and the protein secretion systems

Since *B. pumilus* produces large extracellular proteases that are of interest to the field of biotechnology, the expression of several extracellular proteases was examined. Table 3 showed that the genes for *aprE*, *aprX* and *wprA,* which encode extracellular proteases, were more highly expressed in the stationary growth phase, which was consistent with the activity assay (Fig. 1b). It was notable that the addition of gelatin led to higher expression level of *apr*E at 72 h. However, *aprE* was the major component in the extracellular proteolytic activity in terms of the transcription level. In contrast, the *epr* and *subE* genes were largely expressed at the exponential growth phase (4 h) or the transition phase (12–24 h). However, the expression of *vpr* fluctuated over the entire growth course.

**Table 3:** Relative expression level of the extracellular proteases of *B. pumilus* BA06 during the entirety of each growth phase

Gene ID	Gene	Protein	Medium	Expression level in FPKM value				
				4 h	12 h	24 h	48 h	72 h
peg.2284	*aprE*	serine alkaline protease (AprE)	MM	128	782	8665	18,789	22,894
			GM	71	575	2578	20,671	35,163
peg.658	*aprX*	alkaline serine protease (AprX)	MM	14	42	192	862	4011
			GM	11	40	62	70	4820
peg.1414	*subE*	subtilisin Carlsberg (SubE)	MM	528	437	467	66	37
			GM	376	688	597	220	38
peg.2809	*epr*	Alkaline serine proteinase (Epr)	MM	107	96	76	48	37
			GM	112	115	97	70	42
peg.3435	*vpr*	extracellular protease (Vpr)	MM	879	332	1106	344	703
			GM	747	561	862	1758	801
peg.2794	*wprA*	Wall-associated protease (WprA)	MM	52	185	258	217	316
			GM	97	203	275	198	221

**Fig. 6:**
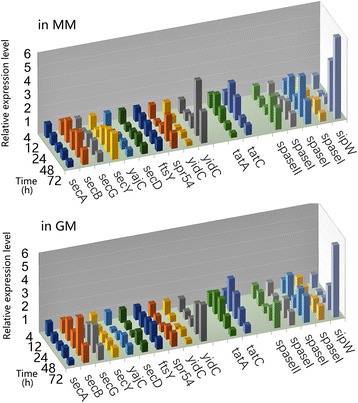
Expression changes in genes involved in the protein secretion pathways of *B. pumilus* BA06 grown in MM and GM during the entire growth curve. The corresponding gene IDs are peg.3162 for *SecA*; peg.2383 for *SecB*; peg.1131 for *SecG*; peg.2418 for *SecY*; peg.1755 for *yajC*; peg.1750 for *SecD*; peg.533 for *ftsY*; peg.535 for *spr54*; peg.3727 for *yidC*; peg.1460 for *yidC*; peg.2558 for *tatA*; peg.2557 for *tatC*; peg.2146 for *Spase II*; peg.2304, peg.364 and peg.482 for *Spase I*, and peg.1525 for *sipW*. The gene expression level (FPKM) of each gene was normalized to 1.00 for the first -time point (4 h)

Protein secretion is generally associated with the *Bacillus* species, which is a critical consideration for the development of the cell factory [42]. By KEGG analysis, two protein secretion pathways were enriched in *B. pumilus* BA06: the Sec-dependent pathway and Tat system. The expression pattern for these two pathways was similar in both MM and GM cultures (Fig. 6 and Additional file 6). However, the major components involved in the Sec-dependent pathway were expressed with two peaks at 4 h and 48 h. In contrast, the expression of the Tat system was increased in the transition phase (12–24 h). Furthermore, various signal peptidases displayed different expression patterns (Fig. 6). These results implied that various secretion systems may function at various growth phases.

### Sigma factors and regulator proteins

**Fig. 7:**
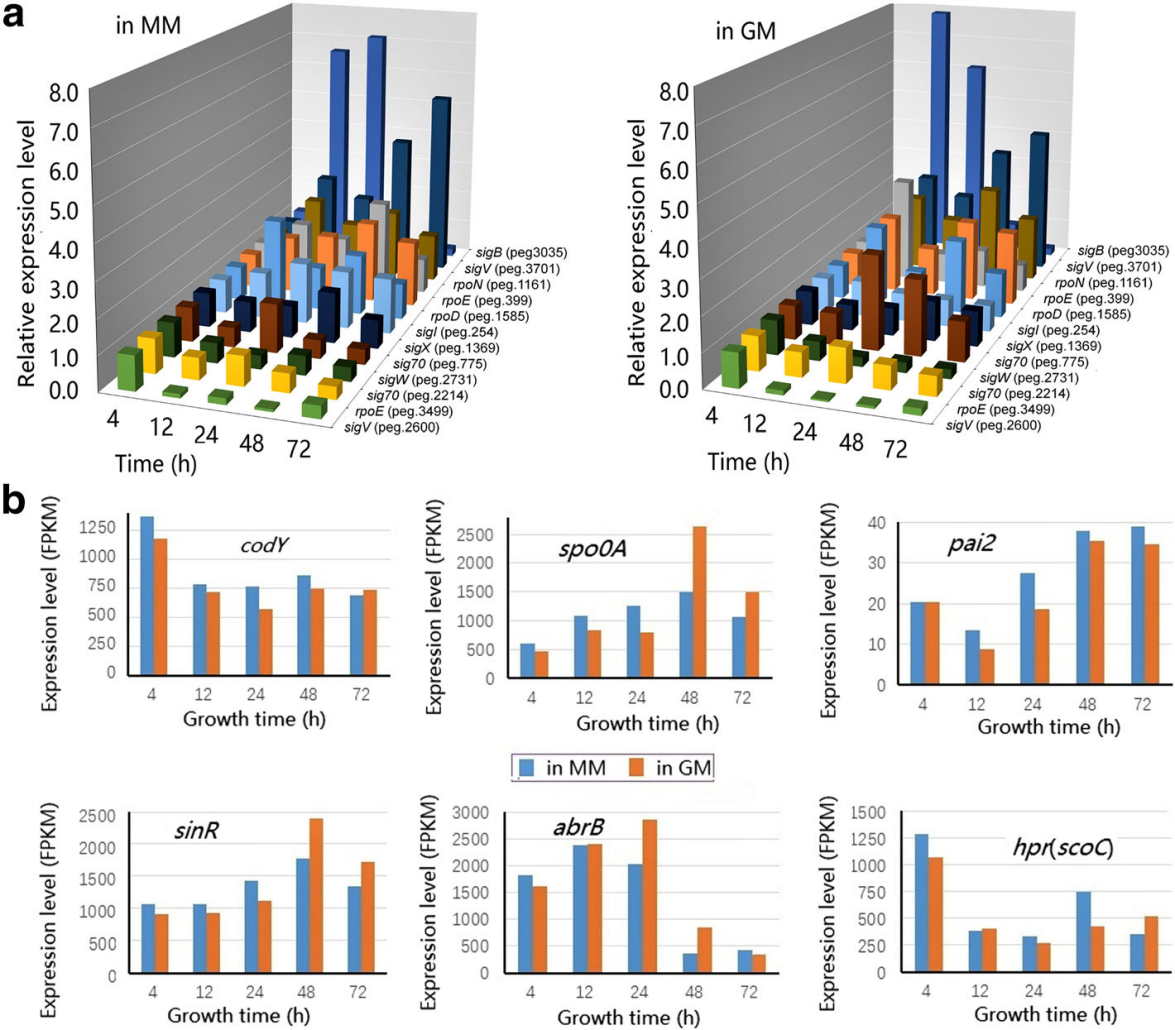
Expression changes in genes encoding sigma factors (**a**) and regulatory proteins (**b**) in *B. pumilus* BA06 grown in MM and GM during the entire growth curve. The gene expression level (FPKM) of each gene was normalized to 1.00 for the first -time point (4 h)

*Bacillus* species usually employ different sigma factors to regulate various physiological processes. For example, SigB in *B. subtilis* mediates the stress response by regulating a large group of genes [43]. Therefore, the expression pattern of the sigma factors was also examined in *B. pumilus* BA06 (Fig. 7a and Additional file 7). The *sigB* gene (peg.3035) displayed a sharp surge in expression in the transition phase (12 h and 24 h), indicating that a stress response may occur at the transition point, which was similar to an observation in *B. subtilis* [44]. Although a SigB regulon in *B. pumilus* was not identified, we expect that a similar regulon may exist in this bacterium.

The other sigma factors exhibited different patterns of expression (Additional file 7). For example, the expression of *sigV* (peg.3701), *rpoN* (peg.1161) and *rpoE* (peg.399) was up-regulated mainly at the stationary growth phase (48 h). In contrast, the expression of *sigV* (peg.2600) and *sig70* (peg.775) was repressed upon entry into the transition phase and thereafter (Fig. 7a).

In *B. subtilis*, several regulatory proteins were recognized as transition-state regulators, such as Hpr (ScoC), AbrB, and SinR. All their homologs were encoded by the BA06 genome. Their expression pattern is shown in Fig. 7b (Additional file 7). The gene for *hpr* was highly expressed at the exponential growth phase (4 h) and then declined. In contrast, expression of *SinR* and *pai2* was increased at 48 h and later, especially in GM. Another transition-state regulator, AbrB, was strongly repressed at 48 h, which is consistent with a similar observation in *B. subtilis* [36].

Global regulators play important roles in the process of gene transcription by binding to the promoter elements. In *Bacillus*, CodY and Spo0A have been recognized as global regulators. Cody, a GTP-binding protein, regulates more than one hundred genes that are typically repressed during rapid (exponential) growth and induced when cells experience nutrient deprivation [45]. Our data indicated that expression of *codY* was higher at the exponential growth phase and then declined. In contrast, the expression of Spo0A, a master regulator of sporulation initiation and secondary metabolism in *B. subtilis* [46], continuously increased up to 48 h. A similar expression pattern for *Spo0A* was also observed in *B. subtilis* [36]*.*

### Validation of the selected DEGs by real-time PCR

To confirm the accuracy and reproducibility of the transcriptome data, 8 genes were selected for qPCR validation. RNA samples from the cultures of MM and GM at different growth phases were used as templates. The data are shown in Additional file 8, indicating that the two sets of data between the RNA-seq and RT-PCR analyses were almost consistent.

## Discussion

In this study, we determined the transcriptome profiles of *B. pumilus* across the various growth phases in both MM and GM cultures. Through DEG analysis and KEGG enrichment, transcriptional changes to genes that are specific to certain metabolic pathways were unveiled at the various growth phases.

During the exponential growth phase, cellular growth is most active along with a quick depletion of nutrients. When the culture enters the transition phase, stress may occur at least through nutrient depletion [47]. At this transition point, a critical alteration in TCA metabolism was revealed in *B. pumilus* BA06. Our results showed that the TCA cycle was most active during the exponential growth phase (Fig. 3). Similarly, TCA -cycle enzymes were frequently identified from the *B. pumilus* cells during the exponential growth phase in the proteomic analysis [16]. The TCA cycle is a central metabolic pathway that not only unifies the carbohydrate, fat and protein metabolic processes, but also produces energy and reducing power. Therefore, the slowdown in the TCA cycle at the transition point and the later growth stage may be a signature of reduced cell activity. Previous studies showed that the expression levels of some genes involved in the TCA cycle decreased when *B. subtilis* was grown under anaerobic conditions [48]. In addition, glucose may regulate the TCA cycle by repressing several genes for the TCA [49]. In fact, at the transition phase and stationary growth phase, glucose in the culture is consumed. Therefore, regulation of the TCA cycle may include other factors. However, secondary metabolism and sporulation will be triggered in the stationary growth phase, which requires the TCA metabolism to maintain carbohydrate flux and energy. Based on our transcriptome data, the carbohydrate flux could be recycled via another branch pathway, from acetyl-CoA to phosphoenol-pyruvate via oxaloacetate, since the genes encoding pyruvate carboxyl transferase (peg.413) and phosphoenolpyruvate carboxykinase (peg.2100) were not repressed but were greatly induced in expression upon entry into the transition phase and later growth phases (Fig. 3).

Another significant change at the transition point is down-regulation of the genes involved in flagellar assembly and the chemotaxis signaling pathway. The flagellum is an organ for mobility, which enables bacterial cells to colonize an ecological niche [50]. Mobility is also regulated by chemotaxis factors [39]. There are several chemoreceptors known as methyl-accepting chemotaxis proteins (MCPs) that are encoded by *B. pumilus*, most of which were revealed as being down-regulated after the exponential growth phase (Additional file 4). However, for other factors, such as surfactin, extracellular proteolytic activity was required to support mobility [51, 52]. The operon *srf* (peg.2905–2910) for biosurfactin biosynthesis was also down-regulated in *B. pumilus* BA06 at the same growth phase (Additional file 4). In fact, *B. pumilus* BA06 could produce surfactant at 24 h of culture, although the yield was lower [53]. Together, these data indicate the attenuation of the swarming mobility of *B. pumilus* upon entering the transition point and thereafter.

Sporulation is a complex cellular process and can be triggered by nutrient limitations [41]. For *B. pumilus* BA06, the formation of endospores was quick during the stationary growth phase (Fig. 1). Meanwhile, our transcriptome data indicated that almost all the genes involved in sporulation continued to be up-regulated after exponential growth up to the stationary growth phase (Fig. 5 and Additional file 5). The addition of gelatin to the MM led to a delay in both sporulation and expression of the sporulation-related genes. However, sporulation is subject to highly hierarchical regulation. In general, the transcriptional factor Spo0A governs the decision to initiate sporulation by phosphorylation via a series of kinases and phosphotransferases [54]. The expression level of *Spo0A* (peg.1484) increased slowly from 4 h to 48 h, which was consistent with a previous study in *B. subtilis* [36]. Since the phosphorylation state is a key factor for Spo0A to initiate sporulation [36], the transcription level observed here may not be relevant to the explanation of its function in sporulation initiation. Five kinases were identified in *B. pumilus*, of which the expression of *KinA* (peg.311, peg.312), *KinC* (peg.373), *KinD* (peg.276), and *KinE* (peg.265) was up-regulated. In contrast, *KinB* (peg.909) was down-regulated at the transition point (Additional file 5). In addition, the sporulation initiation phosphotransferase (Spo0F, peg.3334) was also down-regulated at 12 h and then up-regulated at 48 h and thereafter. Overall, the expression levels of the conserved sporulation kinases and phosphotransferases and Spo0A displayed a pattern that was different from the other sporulation-related genes, whose expression was continuously up-regulated until the later stationary growth phase (Fig. 5). The interruption may be ascribed to their role as only being responsive toward initiating sporulation. Recently, KinD in *B. subtilis* was shown to delay the onset of sporulation [55]. However, the expression level of *KinD* in *B. pumilus* was highest among the five sporulation kinases, which may directly phosphorylate Spo0A [41]. Therefore, KinD may play a more important role in sporulation initiation in *B. pumilus*.

During the sporulation process, the roles of several specific sigma factors SigE, SigF, SigG, and SigK were well documented in regulation of sporulation. These four factors were sequentially activated after sporulation initiation: sigF in the forespore, sigE in the mother cell, sigG in the forespore, and sigK in the mother cell [54]. Our transcriptome data indicated that all four sigma factors were expressed in a similar way with a peak at the later stationary growth phase (Fig. 5). However, another sigma factor, SigH (peg.2382), was found to participate in the regulation of sporulation in *Clostridium difficile* [56]. In *B. pumilus*, *sigH* was expressed in a manner that was different from the above four sigma factors. SigH may regulate the other genes involved in mobility and cell division. Therefore, the *sigH* gene may not be specific to sporulation in *B. pumilus*.

Extracellular protease production and protein secretion are processes of interest to the field of biotechnology. Previously, AprE, Epr, WprA, and Vpr were identified to be extracellular protease in *B. pumilus* SCU11 [57]. Our transcriptome data indicated that the expression patterns of various extracellular proteases were different in *B. pumilus* (Table 3). Three genes (*aprE*, *aprX*, and *wprA*) were expressed more highly during the stationary growth phase. Based on the expression levels, these proteases may contribute to the high extracellular proteolytic activity observed in the culture supernatant. In contrast, *epr* and *subE* were highly expressed in the exponential growth phase (Table 3), which could be explained by the fact that the expression of *epr* was controlled by SigD in *B. subtilis* [58]. However, these results suggested that different regulatory mechanisms and secretion pathways may be employed for the various proteases. In *B. subtilis*, the regulatory mechanism of the *aprE* expression has been extensively studied, indicating that at least two positive regulatory proteins (DegU and Spo0A) are involved in regulation of the *aprE* expression [59, 60]. The expression of *degU* and *spo0A* in *B. pumilus* was up-regulated at 48 h in GM (Additional file 1 and Fig. 7), which may contribute to the observed increase of *aprE* expression in the medium supplemented with gelatin.

Two protein secretion pathways were enriched by KEGG analysis in *B. pumilus*: The Sec-dependent pathway and Tat system, which were also identified by the previous proteomics analysis [16]. The Tat system responds by to secreting twin-arginine (RR/KRP) signal peptides, through which 44 proteins are predicated to be secreted in *B. subtilis* [61]. WprA has been proposed to be secreted through the Tat system in *B. pumilus* [62]. Regarding the expression pattern during the growth course, the Tat system (including TatA and TatC) may respond by secreting limited proteins and may play a major role in the transition phase. In contrast, the large number of genes involved in the Sec secretion pathway are down-regulated at the transition point, and then up-regulated at 48 h. In general, many proteins have been observed to be secreted extracellularly during the stationary growth phase in *B. subtilis* [62]. Therefore, the Sec secretion system may respond by secreting many proteins during the stationary growth phase. For example, AprE, Vpr, and aprX may be secreted via this system [63]. However, 65 proteins were detected from the late exponential growth cultures in *B. pumilus*; this number is much lower than the predicted protein species (513) [57]. Therefore, the Sec-dependent protein secretion system may be the major pathway, especially during the stationary growth phase in *B. pumilus*.

In *Bacillus*, alternative sigma factors are involved in the regulation of certain genes or specific metabolic processes. For example, SigB is generally recognized in response to stress. In *B. subtilis*, approximately 150 general stress-associated genes have been identified as the SigB regulon [64]. The expression of the *sigB* gene (peg.3035) is also up-regulated greatly at the transition phase in *B. pumilus*, which is similar with the observation in *B. subtilis* [37]. Although the sigB regulon has not been identified for *B. pumilus*, the *rsb* operon (peg.3029–3033) is induced upon entry into the transition point (Additional file 9). Other sigma factors such as SigW and SigX have been reported to respond to stress. For example, SigW in *B. subtilis* responds by regulating detoxification and the production of antimicrobial compounds [65]. However, the genes involved in the osmotic and oxidative responses, such as *opuAA* (peg. 2747, peg.3708), *opuAB* (peg.3707), *opuCB* (peg.1139), *opuCA* (peg.1140), *perR* (peg.820), and *cat* (peg.2205, peg.3703), have been shown to be up-regulated at the transition point in *B. pumilus* (Additional file 8: Table S8); some of these genes have also been revealed to be involved in the response to oxidative stress in another study using *B. pumilus* Jo2 and a microarray-based transcriptome analysis [15]. Therefore, *B. pumilus* may suffer from stress when entering the transition phase and subsequently activates a set of stress-related genes to be expressed in a manner that is similar to *B. subtilis*.

## Conclusions

In conclusion, an RNA-seq-based transcriptome analysis was first applied to *B. pumilus* BA06 to monitor the transcriptional profile over the entire growth course in a defined MM or GM medium. Changes to the specific metabolic pathways in terms of gene expression were shown to relate to the transition from the exponential growth phase and the onset of the stationary growth phase. Upon entry into the transition point, one of the striking changes was down-regulation of central TCA metabolism and oxidative phosphorylation. Furthermore, the cellular mobility ability was also reduced because of down-regulation of genes involved in flagellar assembly and the chemotaxis signaling pathway. In contrast, many stress-responding genes including the SigB regulon, were induced in expression after entering the transition point, indicating that the cells may suffer from stress. During the stationary growth phase, a significant change occurred in the genes involved in sporulation, and more than 100 sporulation-related genes were induced, which was consistent with the process of sporulation formation. However, the addition of gelatin to the MM medium did not cause a great impact on the transcriptome profile, except for the genes encoding sporulation-related proteins and extracellular proteases with delayed expression.

## Methods

### Bacterial strain and growth conditions

*B. pumilus* BA06 was routinely maintained on Luria-Bertani (LB, 10 g/l tryptone, 5 g/l yeast extract, 10 g/l NaCl, pH 7.5) agar plate. A single colony of *B. pumilus* BA06 was selected for transfer into 10 ml LB broth and incubated at 37 °C overnight with shaking at 140 rpm. Afterward, 500 μl of an overnight culture was transferred into 50 ml of minimal medium [MM, 1.0 g/l sodium citrate, 2.0 g/l (NH_4_)_2_SO_4_, 14.0 g/l K_2_HPO_4_, 6.0 g/l KH_2_PO_4_, 0.2 g/l MgSO_4_, 2.5 g/l yeast extract, 5.0 g/l D-glucose] and the gelatin-amended minimal medium (GM) (2.0 g/l gelatin) in 250-ml flasks. The cultures were incubated at 37 °C with shaking at 140 rpm for the indicated time points.

At various time points, the cell density was measured by reading the OD_560_ on a spectrometer. The total number of cells and endospores was also determined. To account for the total cell number, a 10-fold dilution of the fresh culture was achieved by serial dilution in sterile PBS buffer. Finally, 0.1 ml of the cell suspension was dispensed onto LB agar plates, and the colonies assigned as the total cells were counted after incubation at 37 °C. To count the number of endospores, an aliquot of fresh culture was sampled and incubated at 55 °C for 15 min to kill the vegetative cells; and the samples were diluted as above and dispensed onto LB agarose plates. The colonies formed on these plates were regarded as endospores. Meanwhile, extracellular protease activity was also assayed using casein as the substrate as described previously [24]. All experiments were performed in triplicate.

### RNA isolation, library construction and Illumina sequencing

The cell samples were pelleted by centrifugation at 8000 rpm at the indicated time points (4, 12, 24, 48, and 72 h) from the *B. pumilus* BA06 cultures in MM and GM, and the cells were then suspended in TE buffer supplemented with 1.5 mg/ml lysozyme and incubated at 37 °C for 10 min, to which the TRIzol reagent (Invitrogen, Invitrogen, Carlsbad, CA) was added. The cell suspension was mixed extensively while using the gauge to disrupt the cells completely. Finally, total RNA was isolated following the instructions provided with the TRIzol reagent. The genomic DNA was removed using the Genome DNA Eraser kit (Takara, Dalian, China).

Subsequently, the rRNA was removed using the Ribo-ZeroTM rRNA Removal kit (Epicentre Biotech, Madison, WI). The resulting mRNAs were fragmented and reverse transcribed using random hexamers as the primer. Second strand cDNA synthesis was performed using DNA Polymerase I and RNase H. The cDNA fragments were processed for end repair and ligated to paired end adaptors. Finally, the library was constructed and sequenced on an Illumina HiSeqTM2000 sequencing platform.

### Mapping and identification of the differentially expressed genes

Clean data were obtained from the raw data by removing the sequences of the adapters and low-quality reads. The clean reads were aligned to the *B. pumilus* genome (GenBank accession number: AMDH00000000) [13] using Bowtie2 with default parameters, allowing up to one-base mismatches [66]. The aligned read files were processed by Cufflinks v2.2.1 [67]. The relative abundances of the transcripts were calculated as the fragments per kilobase of transcript per million fragments mapped (FPKM). Differentially expressed genes (DEGs) among the different samples were extracted by using edgeR in the Bioconductor package [68]. The DEGs were defined with an FDR (false discovery rate) ≤ 0.05 and log_2_fold-change (log_2_FC) ≥ 1.

### Functional classification and KEGG analysis

Functional classification of DEGs was performed online by RAST (Rapid Annotation using Subsystem Technology, http://www.nmpdr.org/FIG/wiki/view.cgi/FIG/ RapidAnnotationServer). RAST is a fully automated service that is especially designed for annotating bacterial and archaeal genomes [35]. Once one gene is annotated, it can be classified into various subsystems.

Meanwhile, the DEGs were further assigned to KEGG (Kyoto Encyclopedia of Genes and Genomes) pathways on the KEGG Automatic Annotation Server (http://www.genome.jp/kegg).

### Quantitative real-time PCR analysis

The expression levels of the selected eight genes (*degS, aprX, glnR, hpr, vpr, sinR, yqkD*, and *spo0A*) of *B. pumilus* BA06 growing under the same conditions, were validated by real-time RT-PCR analysis. The real-time RT-PCR was performed using an iCyclerMyiQ Real-Time PCR System (Bio-Rad, Hercules, CA). The PCR conditions were set up as 95 °C for 2 mins, followed by 40 cycles of 95 °C for 10 s, 65 °C for 15 s and 72 °C for 20 s. A melting curve analysis of the amplification products was performed at the end of each PCR run to ensure that unique products were amplified. The specific primers used for the selected genes are listed in Additional file 10. The expression level was normalized to the internal control gene 16S rRNA, using the 2^−ΔΔCt^ method [69].

## Additional files

Additional file 1: Electrophoresis analysis of RNA samples of *Bacillus pumilus* BA06. (PDF 126 kb)

Additional file 2: The gene annotation by RAST and expression levels (FPKM) across various growth stages of *Bacillus pumilus* BA06. (XLSX 582 kb)

Additional file 3: List of the differentially expressed genes in the tricarboxylic acid cycle and oxidative phosphorylation by KEGG analysis. (XLSX 26 kb)

Additional file 4: List of the differentially expressed genes involved in flagellar assembly and the chemotaxis signaling pathway enriched by KEGG analysis. (XLSX 34 kb)

Additional file 5: List of the differentially expressed genes involved in dormancy and sporulation by RAST analysis. (XLSX 45 kb)

Additional file 6: List of the differentially expressed genes assigned to protein secretion systems by KEGG analysis. (XLSX 17 kb)

Additional file 7: List of the differentially expressed genes encoding regulatory proteins and the sigma factors. (XLSX 19 kb)

Additional file 8: Comparison of expression levels of the selected eight genes between the transcriptome and qPCR methods. (DOCX 17 kb)

Additional file 9: List of the differentially expressed genes assigned as the “Stress Response” subsystem by RAST analysis. (XLSX 37 kb)

Additional file 10: Primers used for real-time PCR. (DOCX 15 kb)
