# Supplementary figures and images for: Transcriptome profiling analysis reveals metabolic changes across various growth phases in Bacillus pumilus BA06
Source: BMC Microbiol. 2017 Jul 11;17:156. doi: 10.1186/s12866-017-1066-7 (PMC5504735; doi:10.1186/s12866-017-1066-7)

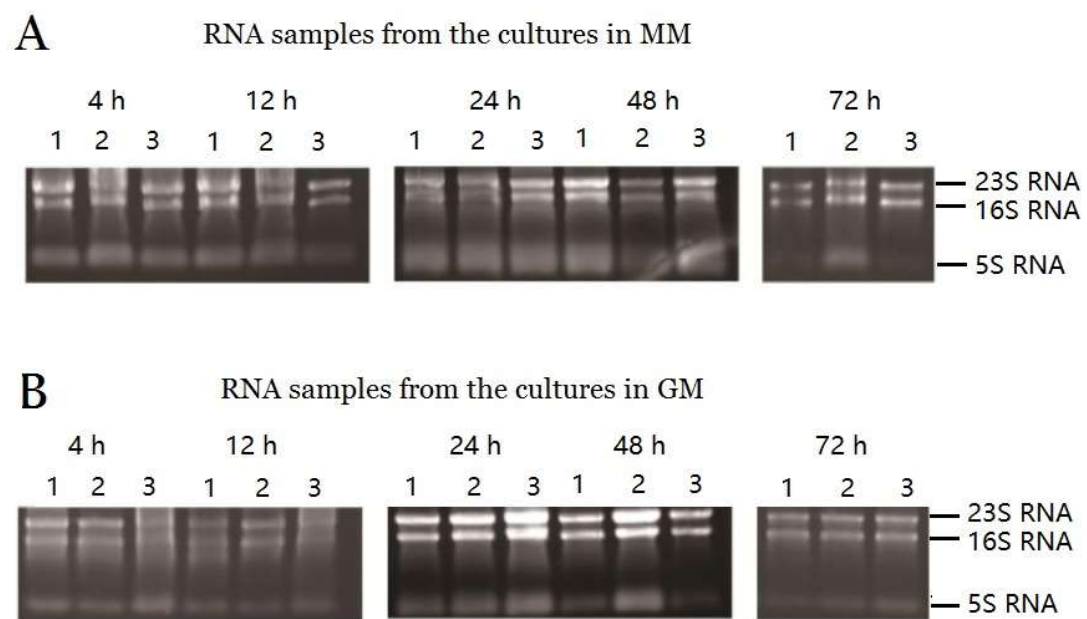

Additional file 1. Electrophoresis analysis of RNA samples of *Bacillus pumilus* BA06

Supplement: Supplementary file 1 — Electrophoresis analysis of RNA samples of Bacillus pumilus BA06. (PDF 126 kb) [file 12866_2017_1066_MOESM1_ESM.pdf]
