# Supplementary material for: Transcriptome profiling analysis reveals metabolic changes across various growth phases in Bacillus pumilus BA06
Source: BMC Microbiol. 2017 Jul 11;17:156. doi: 10.1186/s12866-017-1066-7 (PMC5504735; doi:10.1186/s12866-017-1066-7)
Supplement: Supplementary file 8 — Comparison of expression levels of the selected eight genes between the transcriptome and qPCR methods. (DOCX 17 kb) [file 12866_2017_1066_MOESM8_ESM.docx]

**Additional file 8.** Comparison of expression levels of the selected eight genes between the transcriptome and the qPCR methods

| **Gene name**  **(ID)** | **Function** | **in MM** | | | **in GM** | | |
| --- | --- | --- | --- | --- | --- | --- | --- |
|  |  | **24 h** | **48 h** | **72 h** | **24 h** | **48 h** | **72 h** |
|  |  | Expression level (FPKM) in transcriptome/  Expression level of qPCR normalized to 16s rDNA | | | | | |
| *aprX*  (peg.658) | Protease | 192.12/  0.15 | 862.05/  1.11 | 4010.77/3.78 | 62.34/  0.03 | 69.58/  0.15 | 4819.59/3.98 |
| *Vpr*  (peg.3435) | Protease | 1106.13/1.27 | 344.31/  0.35 | 703.11/  0.49 | 862.19/  0.17 | 1757.88/2.5 | 801.478/0.49 |
| *yqkD*  (peg.1421) | Protease | 827.91/  0.68 | 1752.89/3.19 | 1207.51/1.86 | 508.33/  0.18 | 1277.27/2.46 | 1415.2/  2.04 |
| *glnR*  (peg.247) | Regulator of N utilization | 154.37/  0.70 | 664.89/  3.18 | 43.32/  0.12 | 133.25/  0.14 | 216.84/  0.59 | 187.32/  0.28 |
| *hpr*  (peg.2258) | Regulator of protease | 329.82/  0.52 | 738.65/  3.18 | 353.73/  0.97 | 266.90/  0.35 | 426.50/  2.44 | 511.64/  2.76 |
| *sinR*  (peg.1523) | Regulator of protease | 329.82/  0.58 | 738.65/  1.69 | 353.73/  0.99 | 1124.86/0.23 | 2405.8/  21.52 | 1731.5/  0.89 |
| *degS*  (peg.3183) | Regulator of protease | 610.15/  3.18 | 338.72/  1.53 | 150.26/  0.72 | 706.349/1.96 | 338.89/  0.76 | 249.75/  0.88 |
| *spo0A*  (peg.1484) | Regulator of protease | 1252.73/0.30 | 1486.83/1.93 | 1062.38/0.542 | 798.72/  0.29 | 2627.27/2.52 | 1485.08/0.56 |
