# Supplementary material for: Transcriptome profiling analysis reveals metabolic changes across various growth phases in Bacillus pumilus BA06
Source: BMC Microbiol. 2017 Jul 11;17:156. doi: 10.1186/s12866-017-1066-7 (PMC5504735; doi:10.1186/s12866-017-1066-7)
Supplement: Supplementary file 10 — Primers used for real-time PCR. (DOCX 15 kb) [file 12866_2017_1066_MOESM10_ESM.docx]

| **Gene** | **Forward primer (5’-3’)** | **Reverse primer (5’-3’)** |
| --- | --- | --- |
| *degS* | CTCTGTGAAGGTTGAAGTGACG | CGCTCTTTCATACCAAGTAACC |
| *aprX* | CAAGACGATCCGATGGTGCG | GATCATCATAGGCTCGGACCG |
| *glnR* | GTCATTTCAATCGGGATTGTCAG | CGTGTGCCTCTTGTTGAGCG |
| *hpr* | CAGCCGCTTCATGACATTTAC | CCTCTGCTGAGTCTTCAAGTTC |
| *vpr* | ACCTTCGCTCGTGCGTCAC | ACCTTCGCTCGTGCGTCAC |
| *sinR* | GGCTACTCACTATCAGAACTGG | AACAGTGTATGAACCGAGACG |
| *yqkD* | TCGATACAGAAGCTGATCCTC | ACACGGAGAATCGATGGGAG |
| *spo0A* | AAGAGCCCGACGTTCTCCTC | CGTCAGCATAATGACACTTGGC |
| *16S rRNA* | AGCCGCGGTAATACGTAGG | TCCACTCTCCTCTTCTGCAC |

**Additional file 10.** Primers used for the real-time PCR
